# The impact of COVID-19-related quarantine on psychological outcomes in patients after cardiac intervention: a multicenter longitudinal study

**DOI:** 10.1038/s41398-022-01984-0

**Published:** 2022-06-06

**Authors:** Elisabetta Patron, Simone Messerotti Benvenuti, Andrea Ponchia, Franco Del Piccolo, Claudio Gentili, Daniela Palomba

**Affiliations:** 1 Department of General Psychology, University of Padua, Padua, Italy; 2 Padova Neuroscience Center (PNC), University of Padua, Padua, Italy; 3 Unit of Cardiac Rehabilitation, ULSS 6 Euganea, Padua, Italy; 4 Unit of Cardiac Rehabilitation, San Marco Hospital, Venice, Italy

**Keywords:** Psychology, Depression

## Abstract

Mandatory quarantine during the COVID-19 pandemic had substantial negative consequences on psychological health in the general population. Depression, anxiety, and insomnia were reported to increase the morbidity and mortality risk in cardiac patients after cardiac interventions. Nonetheless, a gap in the evidence appeared regarding the effects of COVID-19-related quarantine on psychological outcomes in patients after cardiac interventions. The present study aimed to longitudinally investigate the effects of quarantine on depressive, anxiety, and insomnia symptoms in a group of patients who underwent cardiac intervention. Seventy-three patients admitted for cardiac rehabilitation completed a psychological assessment before and a reassessment after the quarantine and were included in the quarantine group. The control group included 76 patients who completed both evaluations before the quarantine. Depressive (Beck Depression Inventory-II; BDI-II), anxiety (Beck Anxiety Inventory-II; BAI), and insomnia (Sleep Condition Indicator; SCI) symptoms were evaluated in both groups at one (assessment) and eight (reassessment) months after cardiac intervention. The statistical analyses revealed that at reassessment, the quarantine group showed higher global depressive, anxiety, and insomnia symptoms than the control group and increased cognitive symptoms of depression. A higher presence of clinically relevant depressed patients was seen in the quarantine group. The present results showed that the COVID-19-related mandatory quarantine negatively affected psychological outcomes in patients after cardiac intervention, increasing the probability for these patients to be depressed. This, in turn, could influence patients’ health in a critical period for morbidity and mortality risk. This underlines the priority of integrating and improving targeted mental health support as the pandemic continues, especially for cardiac patients.

## Introduction

Italy was severely affected by the first wave of the coronavirus disease 2019 (COVID-19) outbreak in 2020, and the population was subjected to a mandatory quarantine for almost 2 months to prevent, or minimize, the impact of the outbreak [1]. The quarantine had negative consequences on psychological outcomes, including depression [2, 3], anxiety [4, 5], eating disorders [6], and insomnia [7, 8]. In Italy, during the quarantine, an increased percentage of people with very high levels of distress was reported compared to epidemiological data from before the pandemic [2].

Cardiac patients are at higher risk of developing severe COVID-19-related complications and show a higher fatality rate (10.5%) than the general population (2.3%) [9]. Even cardiac patients who were not directly affected by COVID-19 suffered from the consequences of isolation during the quarantine. Specifically, a cross-sectional study reported a high prevalence of depressive and anxiety symptoms among postmyocardial infarction patients during the COVID-19 pandemic [10]. Moreover, a prospective study with chronic cardiovascular diseases reported worse health-related quality of life during the COVID-19 outbreak, including increased anxiety and depression [11].

This is of paramount relevance considering that a strong bidirectional relationship between psychological distress (such as depressive, anxiety, and insomnia symptoms) and cardiac disease onset and/or mortality has been consistently reported [12]. Depression, anxiety, and insomnia are often reported after cardiac intervention [13] and have been found to increase the risk for morbidity and mortality independent of medical factors [14]. In the context of the COVID-19 pandemic, the burden of social isolation due to the mandatory quarantine could have had an additional effect on common postoperative depressive, anxiety, and insomnia symptoms. This, in turn, could have further undermined the recovery process in cardiac patients who underwent cardiac intervention. To date, however, no study has evaluated the effects of social isolation due to mandatory quarantine during the COVID-19 pandemic on psychological outcomes in cardiac patients after cardiac intervention.

In light of these considerations, to our knowledge, this is the first multicenter longitudinal study that aimed to investigate the effects of quarantine on psychological outcomes in cardiac patients who underwent a cardiac intervention before the COVID-19 outbreak and were affected by quarantine policies in the critical timeframe between six and 12 months after the cardiac intervention. To evaluate the effects of quarantine on psychological outcomes, cardiac patients who had experienced the quarantine was compared to cardiac patients who had completed the study (assessment and reassessment) before the COVID-19 outbreak. It was hypothesized that patients who experienced quarantine in the critical period after a cardiac intervention would show higher depressive, anxiety, and insomnia symptoms than those in the control group.

## Methods

### Participants

Consecutive patients who were referred for a cardiovascular examination after a cardiac intervention to the Unit of Cardiac Rehabilitation, ULSS 6 Euganea, (Padua, Italy), or to the Unit of Cardiac Rehabilitation, San Marco Hospital (Venice, Italy) between December 2017 and January 2020 were asked to participate in the study. Of the 447 patients approached, 58 (13%) were unable to take part in the study, and 10 (2%) declined participation.

Patients in the control group completed the reassessment in the same hospital setting as their assessment before the COVID-19 outbreak in Italy (the last reassessment occurred on 3 December 2019). Patients in the quarantine group completed the reassessment through an online-based form (between 4th May and 18th September 2020) due to the recommended restrictions in hospital routine operations after the COVID-19 outbreak. In the two provinces where the study was carried out (Venice and Padua Provinces in northeastern Italy), a strict lockdown or quarantine was imposed beginning on 8th March 2020, which restricted the movement of the population except for obtaining necessities, work, and health circumstances. During the lockdown, individuals were not allowed to leave their houses to visit relatives or other loved ones, bars, restaurants, or nonauthorized shops, and parks and public gardens were closed. There was a gradual easing of the restrictions beginning on 4 May 2020. People were allowed to leave their houses to visit family and to perform physical activity and some nonessential activities, and some public parks and gardens reopened [15].

The exclusion criteria were as follows: an inability to read or understand Italian; visual or auditory impairments; participation in a conflicting research protocol; a life-threatening condition; and a history of severe psychiatric illness, and/or symptomatic cerebrovascular disease and/or neurological deficits as obtained from a patient’s medical records and confirmed by medical staff.

The study was conducted in accordance with the Declaration of Helsinki, and all procedures were performed with the patients’ adequate understanding and written consent. This study was part of a larger research project conducted at the Unit of Cardiac Rehabilitation, ULSS 6 Euganea (Padua, Italy) and the Unit of Cardiac Rehabilitation, San Marco Hospital (Venice, Italy), which was approved by the local ethics committees (Nucleo di Ricerca Clinica - AULSS 6 Euganea, Prot. No. 209498; Comitato Etico Sperimentazione Clinica Provincia Di Venezia e IRCSS San Camillo (CESC), Prot. No. 5137B6558BA9E00C7BE4CBFD4FED0BFA; Comitato Etico Della Ricerca Psicologica (AREA 17), Prot. No. 2229).

### Assessment of demographic, cardiac risk, biomedical, and psychological variables

The assessment was performed after the cardiovascular examination in a quiet and isolated room at one of the hospitals included in the research project. A short semistructured interview and three questionnaires were administered individually by a trained psychologist. The semistructured interview was administered only during the assessment and allowed for the collection of information on demographic variables (age, sex, and years of education), the type of cardiac intervention [i.e., surgery, including coronary artery bypass graft (CABG), cardiac valve replacement or repair; and percutaneous transluminal coronary angioplasty (PTCA)], days since the cardiac intervention, cardiac risk factors (i.e., hypertension, atrial fibrillation, diabetes, dyslipidemia), medication (i.e., β-blockers, antihypertensives, antiarrhythmics, anticoagulants, ACE-inhibitors), and the total minutes the patient spent walking during the previous week. Systolic (SBP) and diastolic (DBP) blood pressure and body mass index (BMI) were obtained from the patients’ most recent medical records. The age-adjusted Charlson Comorbidity Index (CCI) [16] scores were calculated through the patients’ most recent medical records. The CCI is a weighted index that accounts for the number and seriousness of comorbid diseases, which may affect mortality risk [17, 18]. The CCI includes 19 medical conditions (e.g., cerebrovascular disease, dementia, or diabetes), with total scores ranging from 0–37 and higher scores indicating greater and more severe medical comorbidities. An automated program by Hall and colleagues was used to calculate the CCI scores [19]. The presence of depressive, anxiety, and insomnia symptoms was assessed by employing:

1. the Beck Depression Inventory-II (BDI-II) was used to evaluate the severity of depressive symptoms in the last two weeks [20, 21]. It includes a cognitive (BDI-II cognitive) and a somatic-affective (BDI-II somatic) subscale. Scores above 13 indicate clinically relevant depressive symptoms [21].
2. The Beck Anxiety Inventory (BAI) [22, 23] was used to evaluate anxiety symptoms. Scores above 7 reflect clinically relevant anxiety symptoms [23].
3. The Sleep Condition Indicator (SCI) [24] is a screening scale that is compliant with the Diagnostic and Statistical Manual of Mental Disorders - Fifth Edition (DSM-5) and was used to evaluate sleep problems and insomnia in the last month. Scores below 16 indicate the minimum criteria for a putative insomnia disorder [24].

### Procedure

Patients were assessed after the cardiovascular examination, on the same day of admission for cardiac rehabilitation (assessment). Specifically, each assessment took place at the Unit of Cardiac Rehabilitation, ULSS 6 Euganea, (Padua, Italy), or San Marco Hospital (Venice, Italy), ~1 month [mean (SD) of 28.28 (19.54) days] after the cardiac intervention. Psychological outcomes, including depressive, anxiety, and insomnia symptoms, were assessed at 1 month (assessment) and ~8 months (reassessment) after the cardiac intervention.

The reassessment was completed approximately eight months after the assessment [mean (SD) of 8.07 (2.94) months], and only the questionnaires (i.e., the BDI-II, BAI, and SCI) were administered. At the time of reassessment, all patients had completed the cardiac rehabilitation protocol. The timeframe for reassessment was chosen because in the period from six to 12 months after cardiac intervention, depressive, anxiety, and insomnia symptoms that might occur in the acute postintervention phase (i.e., in the first 4 weeks) have been reported to generally decrease and stabilize [25].

### Data reduction and statistical analysis

To determine the sample size required to estimate the effect with an adequate level of precision, a power analysis was performed using GPower 3.1 [26]. Since using the effect sizes of published articles as an estimate for power analysis has been reported as bad practice in clinical psychology, a small effect size (*η*² = 0.03) was assumed [27]. The total number of participants needed for 80% power in a repeated measures design with a small effect size (*η*² = 0.03) was 108 participants. Therefore, the sample of the present study was considered adequate.

Patients in the quarantine and control groups were compared in terms of all the variables collected through the semistructured interview or obtained from the patients’ most recent medical records. Specifically, Student’s *t* tests for continuous variables and *χ*^2^ tests for categorical values were performed.

To control for differences between the patients included in the study and those not included (i.e., a total of 190 patients who were unable to complete the protocol or declined to continue participating), the two groups were compared for all variables collected at assessment.

To evaluate and quantify the effect of quarantine on psychological outcomes, mixed model repeated measure analyses were applied (i.e., the BDI-II, BAI, and SCI), controlling for the type of cardiac intervention (surgery, PTCA), age-adjusted Charlson Comorbidity Index (CCI) scores, and days since cardiac intervention, and including time (two level factor: assessment and reassessment), group (two level factor: quarantine and control), and time × group interactions (four level: quarantine group at assessment, quarantine group at reassessment, control group at assessment and control group at reassessment) as fixed effects. The subject was included as a random effect. The results from the analyses (i.e., all comparisons between the groups and mixed models) were considered significant if they survived Holm–Bonferroni correction for multiple comparisons. Significant interactions (*p* < 0.05) were followed by Tukey post hoc comparisons to identify specific differences and to determine the exact nature of the interactions.

To test whether the quarantine influenced the number of patients with depressive, anxious, and insomnia symptoms, the presence of clinically relevant depression (coded as 0 = absent for BDI-II scores ≤ 13; 1 = present for BDI-II scores;> 13), anxiety (coded as 0 = absent for BAI scores ≤ 7; 1 = present for BAI scores > 7), and insomnia (coded as 0 = absent for SCI scores ≥ 16; 1 = present for SCI scores < 16) was calculated and compared by applying separate *χ*^2^ tests at assessment and reassessment. When significant effects emerged from the *χ*^2^ tests, the quarantine influence on the number patients with depressive, anxious, and insomnia symptoms was examined through a logistic regression model predicting the presence (of depression, anxiety, or insomnia symptoms) at reassessment, including as predictors in the first block (the presence of depression, anxiety or insomnia symptoms at assessment) and the second block (the quarantine and control groups).

All analyses were performed using R (version 3.6.1, R Development Core & Team, 2011).

## Results

### Characteristics of the patients in the study

**Fig. 1:**
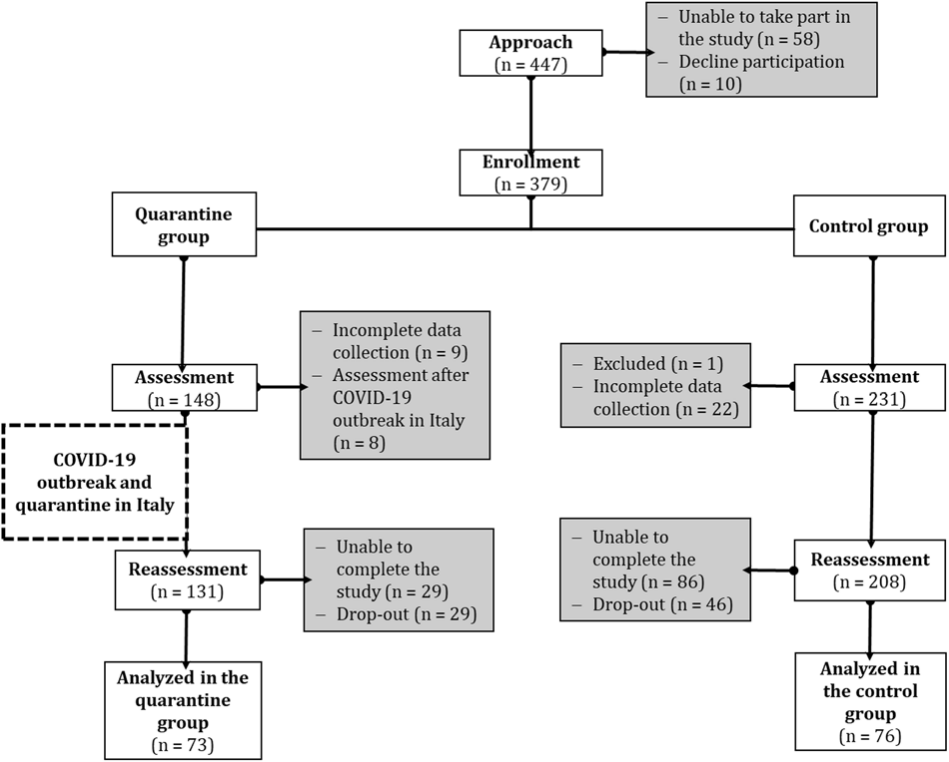
STROBE diagram ofpatient enrollment in the study. Details information on patient enrollment throughout the study, including patients in the Quarantine and the Control groups. STROBE, Strengthening the reporting of observational studies in epidemiology.

Figure 1 summarize patients selection procedure. Of the 379 patients recruited, 148 (39%) were included in the quarantine group, and 231 (61%) were included in the control group. Of the patients assessed in the quarantine group, data collection was incomplete for nine (6%) patients, and eight (5%) patients were excluded since the assessment collection occurred after the outbreak of COVID-19 in Italy (see Fig. 1). One hundred thirty-one patients in the quarantine group completed the assessment and were contacted after the quarantine for reassessment. Of these patients, 29 (21%) could not be contacted, and 29 (21%) declined to continue participating. The quarantine group consisted of 73 analyzed patients, mostly men (*n* = 60, 82%), with a mean [standard deviation (SD)] age of 62.71 (10.22) years and a mean (SD) education of 12.38 (4.22) years. None of the patients in the quarantine group reported having suffered from COVID-19 or having tested positive for COVID-19 during the study. Of the patients in the control group, one was excluded for the inability to read or understand Italian, and 22 patients had incomplete data collection. Two hundred-eight patients met the inclusion criteria, completed the assessment, and were contacted for reassessment. Of these patients, 86 (41%) could not be contacted or had moved outside the area, and 46 (22%) declined to continue participating. The control group consisted of 76 analyzed patients, mostly men (*n* = 69, 91%), with a mean (SD) age of 61.28 (10.23) years and a mean (SD) education of 13.07 (4.32) years.

No differences emerged between the patients in the quarantine and control groups for the demographic variables, days since the cardiac intervention, type of intervention, cardiac risk factors including CCI scores, medication, SBP, DBP, BMI, and walking time in the previous week (all *p* > 0.127; see Table 1).

**Table 1:** Characteristics of the patients enrolled in the study.

	Quarantine group	Control group	t/χ^2^	*p*
	(*n* = 73)	(*n* = 76)		
Demographic characteristics				
Age (years)	62.71 (10.22)	61.28 (10.23)	−0.86	0.393
Sex, male (*N*, %)	60 (82)	69 (91)	1.69	0.194
Education (years)	12.38 (4.22)	13.07 (4.32)	0.97	0.331
Type of cardiac intervention			2.33	0.127
Surgery (*N*, %)	20 (27)	12 (16)	–	–
Procedure (*N*, %)	53 (73)	64 (84)	–	–
Days since surgery	33.52 (39.97)	26.64 (19.08)	−1.33	0.186
Cardiac risk factors				
Hypertension (*N*, %)	56 (77)	54 (71)	0.36	0.549
Atrial fibrillation (*N*, %)	20 (27)	17 (22)	0.27	0.603
Diabetes (*N*, %)	11 (15)	12 (16)	0.001	0.999
Dyslipidemia (*N*, %)	39 (53)	45 (59)	0.30	0.585
CCI score	2.70 (1.25)	2.59 (1.34)	−0.50	0.617
Medications				
β-blockers (*N*, %)	57 (78)	64 (84)	0.56	0.455
Antihypertensives (*N*, %)	29 (40)	23 (30)	1.08	0.299
Antiarrhythmics (*N*, %)	14 (19)	7 (9)	2.29	0.130
Anticoagulants (*N*, %)	71 (97)	76 (100)	0.55	0.459
ACE-inhibitors (*N*, %)	31 (42)	39 (51)	0.84	0.359
Psychiatric drugs (*N*, %)	4 (5)	2 (3)	0.22	0.640
Biomedical and behavioral characteristics				
Systolic Blood Pressure (mmHg)	127.40 (15.48)	129.71 (14.87)	0.85	0.394
Diastolic Blood Pressure (mmHg)	77.37 (7.45)	77.79 (7.13)	0.33	0.744
BMI (kg/m^2^)	26.10 (2.94)	26.64 (3.59)	0.99	0.323
Walking (total minutes in the last week)	194.52 (185.56)	242.93 (199.49)	1.53	0.127

Data are the *M* (SD) of continuous variables and the *N* (%) of categorical variables.

*BMI* body mass index, *CCI* age-adjusted Charlson Comorbidity Index, *ACE- inhibitors* angiotensin-converting-enzyme inhibitors.

Patients included in the study had higher education levels [mean (SD) education of 12.73 (4.27) years] than the patients who were not included [mean (SD) of 11.00 (4.36) years; *t* = −3.67; *p* < 0.001] (see Table 1 and Supplementary material). No other difference between the patients who were included and not included in the study survived Holm–Bonferroni correction for multiple comparisons.

### Effects of the COVID-19-related quarantine on psychological variables

#### Depressive symptoms

**Fig. 2:**
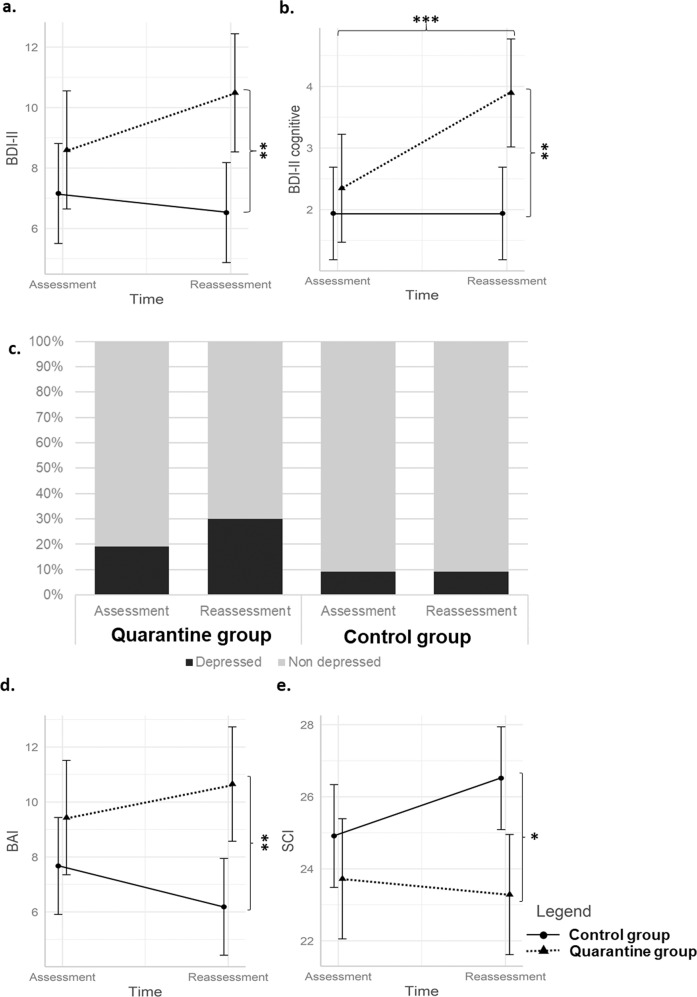
Effects of the COVID-19-related quarantine on psychological variables. **a** BDI-II scores in the quarantine and control groups at assessment and reassessment. **b** BDI-II cognitive scale scores in the quarantine and control groups at assessment and reassessment. **c** Presence of depression in the quarantine and control groups at assessment and reassessment (coded as 0 = nondepressed for BDI-II scores ≤ 13; 1 = depressed for BDI-II scores > 13). **d** BAI scores in the quarantine and control groups at assessment and reassessment. **e** SCI scores in the quarantine and control groups at assessment and reassessment. BDI-II Beck Depression Inventory II, BAI Beck Anxiety Inventory, SCI Sleep Condition Indicator. Error bars represent the 95% confidence intervals. *Tuckey Post hoc *p* < 0.05; **Tuckey Post hoc *p* < 0.01; ***Tuckey Post hoc *p* < 0.001.

The mixed model on BDI-II scores showed a time × group interaction effect (*β* = 2.52; 95% C.I.: 0.45–4.59; *p* = 0.018; *η*² = 0.04; see Tables 2 and 3). Tuckey post hoc revealed that the quarantine group showed higher depressive symptoms than the control group at reassessment (*p* = 0.005; see Fig. 2a). No main time or group effects emerged (see Table 3). On the BDI-II somatic subscale, no time × group interaction or main time or group effects emerged (see Table 3). The mixed model on the BDI-II cognitive subscale revealed a significant time × group interaction effect (*β* = 1.55; 95% C.I.: 0.52–2.57; *p* = 0.004; *η*² = 0.06). The quarantine group had higher cognitive depressive symptoms than the control group at reassessment (*p* = 0.003). Additionally, the quarantine group showed a significant increase in cognitive depressive symptoms from assessment to reassessment (*p* < 0.001; see Fig. 2b). No main time or group effects emerged for the BDI-II cognitive subscale.

**Table 2:** Psychological variables of the patients enrolled in the study.

	Quarantine group		Control group	
	(*n* = 73)		(*n* = 76)	
	Assessment	Reassessment	Assessment	Reassessment
BDI-II score	8.56 (6.03)	10.45 (8.87)	7.24 (6.23)	6.60 (6.82)
BDI-II somatic score	6.30 (4.39)	6.78 (5.37)	5.20 (4.29)	4.57 (4.20)
BDI-II cognitive score	2.26 (2.28)	3.81 (4.39)	2.04 (2.82)	2.04 (3.10)
BAI score	9.29 (7.33)	10.51 (8.89)	7.64 (7.16)	6.16 (6.12)
SCI score	23.77 (5.81)	23.33 (6.69)	24.71 (6.22)	26.32 (5.76)

Data are the *M* (SD) of the variables.

*BDI-II* Beck Depression Inventory II; *BAI* Beck Anxiety Inventory; *SCI* Sleep Condition Indicator.

**Table 3:** Repeated measures mixed models in the quarantine and control groups from assessment to reassessment.

BDI-II	β	SE	df	95% C.I.		t	η²	p value
Intercept	9.42	2.05	153.68	5.45	13.39	4.60	–	<0.001***
Intervention (surgery-PTCA)	0.62	1.32	144.00	−1.94	3.19	0.47	0.001	0.638
CCI score	−0.44	0.42	144.00	−1.256	0.368	−1.06	0.008	0.291
Days between evaluations (assessment-reassessment)	−0.005	0.006	144.00	−0.017	0.007	−0.83	0.005	0.406
Time	−0.63	0.74	147.00	−2.08	0.81	−0.85	0.01	0.394
Group	1.57	1.22	207.21	−0.80	3.93	1.28	0.04	0.200
Time × group	2.52	1.05	147.00	0.45	4.59	2.39	0.04	0.018*

BDI-II somatic	β	SE	df	95% C.I.		t	η²	*p* value
Intercept	5.71	1.32	154.05	3.15	8.28	4.32	–	<0.001***
Intervention (surgery-PTCA)	0.91	0.85	144.00	−0.75	2.56	1.06	0.01	0.290
CCI score	−0.009	0.27	144.00	−0.53	0.52	−0.03	0.0001	0.973
Days between evaluations (assessment-reassessment)	−0.003	0.004	144.00	−0.01	0.005	−0.72	0.004	0.472
Time	−0.63	0.49	147.00	−1.58	0.32	−1.23	0.0003	0.196
Group	1.15	0.79	209.363	−0.38	2.68	1.45	0.04	0.148
Time × group	1.11	0.69	147.00	−0.25	2.47	1.60	0.02	0.112

BDI-II cognitive	β	SE	df	95% C.I.		t	η²	*p* value
Intercept	3.73	3.73	156.30	1.97	5.48	4.12	–	<0.001***
Intervention (surgery-PTCA)	−0.37	−0.37	144.00	−1.50	0.75	−0.64	0.003	0.523
CCI score	−0.47	−0.47	144.00	−0.83	−0.12	−2.57	0.04	0.011*
Days between evaluations (assessment-reassessment)	−0.002	−0.002	144.00	−0.01	0.003	−0.67	0.003	0.505
Time	0.0001	0.0001	147	−0.72	0.72	0.0001	0.06	0.999
Group	0.409	0.409	221.90	−0.66	1.48	0.744	0.04	0.458
Time × group	1.55	1.55	147.00	0.52	2.57	2.96	0.06	0.004**

BAI	β	SE	df	95% C.I.		t	η²	*p* value
Intercept	8.49	2.22	150.61	4.18	12.79	3.82	–	<0.001***
Intervention surgery-PTCA)	−1.25	1.44	144.00	−4.04	1.54	−0.87	0.005	0.386
CCI score	−0.360	0.456	144.00	−1.24	0.52	−0.79	0.004	0.432
Days between evaluations (assessment-reassessment)	0.001	0.01	144.00	−0.01	0.01	0.19	0.0003	0.847
Time	−1.49	0.66	147.00	−2.79	−0.18	−2.23	0.0005	0.027*
Group	1.76	1.29	188.29	−0.74	4.26	1.36	0.04	0.174
Time × group	2.71	0.95	147.00	0.84	4.57	2.85	0.05	0.005**

SCI	β	SE	df	95% C.I.		t	η²	*p* value
Intercept	22.76	1.74	154.73	19.39	26.14	13.09		<0.001***
Intervention (surgery-PTCA)	−1.90	1.12	144.00	−4.07	0.27	−1.69	0.02	0.092
CCI score	0.13	0.35	144.00	−0.56	0.81	0.36	0.001	0.722
Days between evaluations (assessment-reassessment)	0.01	0.005	144.00	−0.001	0.02	1.66	0.02	0.099
Time	1.60	0.66	147.00	0.31	2.90	2.43	0.01	0.016*
Group	−1.19	1.04	213.31	−3.21	0.84	−1.14	0.04	0.257
Time × group	−2.04	0.94	147.00	−3.89	−0.20	−2.17	0.03	0.032*

*CC* age-adjusted Charlson Comorbidity Index, *BDI-II* Beck Depression Inventory II, *BAI* Beck Anxiety Inventory, *SCI* sleep condition indicator. *p < 0.05 **p < 0.01; ***p < 0.001.

Regarding the presence of clinically relevant depression, at assessment, 14 (19%) patients in the quarantine group and 7 (9%) in the control group were depressed (BDI-II scores > 13 coded as 1), and no difference emerged (*χ*^2^ = 2.29; *p* = 0.130). At reassessment, 22 (30%) patients in the quarantine group and 7 (9%) patients in the control group were depressed, and the *χ*^2^ test yielded a significant difference (*χ*^2^ = 9.11; *p* = 0.002; see Fig. 2c). The final model of the regression showed that the group (quarantine vs. control) significantly predicted the presence of depression at reassessment (*β* = 1.43; SE = 0.55; *Z* = 2.61; OR = 4.20; *p* = 0.009) after controlling for the presence of depression at assessment (*β* = 3.00; SE = 0.59; *Z* = 5.06; OR = 20.18; *p* < 0.001). The inclusion of the group (quarantine vs. control) added 5% explained variance to the model (*χ*^2^ = 7.70, *p* = 0.006).

#### Anxiety symptoms

The mixed model on BAI scores revealed a significant time effect (*β* = −1.49; 95% C.I.: −2.79 to −0.18; *p* = 0.027; *η*² = 0.0005), which was qualified by a significant time × group interaction (*β* = 2.71; 95% C.I.: 0.84–4.57; *p* = 0.005; *η*² = 0.05). The quarantine group had significantly higher anxiety symptoms than the control group at reassessment (*p* = 0.004; see Fig. 2d). No main effect for group emerged (see Table 3). Concerning the presence of clinically relevant anxiety, at assessment, 37 (51%) patients in the quarantine group and 35 (46%) patients in the control group were anxious (BAI score > 7 coded as 1), and no differences emerged (*χ*^2^ = 0.16; *p* = 0.688). At reassessment, 39 (53%) patients in the quarantine group and 29 (38%) patients in the control group were anxious, and no significant difference emerged (*χ*^2^ = 2.91; *p* = 0.088).

#### Sleep

The mixed model on SCI scores showed a significant time effect (*β* = 1.60; 95% C.I.: 0.31–2.90; *p* = 0.016; *η*² = 0.01) that was qualified by the time × group interaction (*β* = −2.04; 95% C.I.: −3.89 to −0.20; *p* = 0.032; *η*² = 0.03). Specifically, the quarantine group had significantly lower sleep quality than the control group at reassessment (*p* = 0.012; see Fig. 2e). No main effect for group emerged (see Table 3). Regarding the presence of clinically relevant insomnia, at assessment, 10 (14%) patients in the quarantine group and 6 (8%) patients in the control group had insomnia (SCI scores <16 coded as 1), and no difference emerged (*χ*^2^ = 0.77; *p* = 0.379). At reassessment, 13 (18%) patients in the quarantine group and 6 (8%) patients in the control group had insomnia, and no significant difference emerged (*χ*^2^ = 2.46; *p* = 0.117).

## Discussion

To our knowledge, this is the first multicenter longitudinal study evaluating the impact of COVID-19-related quarantine on psychological outcomes in cardiac patients who underwent a cardiac intervention before the COVID-19 outbreak and were affected by quarantine policies in the critical timeframe between six and 12 months after the cardiac intervention. In line with data on the general population [28–30] and on chronic patients [11, 31], the present results indicated that cardiac patients who experienced the mandatory quarantine during the COVID-19 pandemic exhibited higher overall depressive, anxiety, and insomnia symptoms at reassessment compared to controls. The present results are in line with those of a prospective study showing the negative effects of COVID-19-related quarantine on the health-related quality of life of cardiac patients [11]. Moreover, a significant increase in cognitive symptoms of depression emerged in patients who experienced the quarantine. In the present study, quarantine was linked to an increased presence of clinically relevant depression in the present sample. Cardiac patients who experienced the quarantine were 4.2 times more likely to show clinically relevant depressive symptoms than cardiac patients who did not experience the quarantine.

This is of paramount relevance when considering that worse outcomes and higher mortality have been independently associated with elevated depressive [32], anxiety [33], and insomnia symptoms in cardiac patients [34]. Moreover, depression after cardiac intervention has been associated with adverse cardiovascular outcomes, higher rehospitalization, and mortality [35].

Cardiac rehabilitation programs after cardiac intervention represent an important step aimed at reducing cardiovascular risk through multidisciplinary interventions, including individualized exercise training, education on nutrition, stress management, cardiovascular risk factor management, and pharmacological treatment optimization. Cardiac rehabilitation programs usually support patient recovery by reducing the risk of rehospitalization and mortality [36]. Nonetheless, depression, anxiety, and insomnia symptoms have been shown to undermine the effectiveness of cardiovascular rehabilitation after cardiac intervention [37–39].

The role of depressive, anxiety, and insomnia symptoms on adverse outcomes has been explained through biological and behavioral mechanisms. Hypothalamic–pituitary–adrenal axis hyperactivity [40], autonomic nervous system imbalances [41], altered inflammatory responses [42], and high platelet aggregability [43] have been considered the most important biological mechanisms underlying the relationship between depression, anxiety, and insomnia symptoms, and increased cardiac risk. Among behavioral mechanisms, smoking, poor physical activity, poor dietary habits, and, more importantly, low adherence to treatment, have been reported [44]. Not only do patients with affective disorders frequently show comorbid conditions such as obesity, hypercholesterolemia, hypertension, and diabetes, but they are also less likely to comply with prescribed medications [45] and are less adherent to physical exercise and smoking cessation programs [46]. Intriguingly, recent meta-analyses of genome-wide association studies and candidate gene studies recognized shared genetic architecture and common genetic mechanisms in mood disorders and cardiovascular diseases [47–49].

In the context of the COVID-19 pandemic, the burden of social isolation due to the mandatory quarantine seems to have increased depressive, anxiety, and insomnia symptoms, which, in turn, could have hindered the positive effects of cardiac rehabilitation programs, leading to higher cardiac risk. It should be noted that none of the patients included in the present study reported suffering from COVID-19 or having tested positive for COVID-19 during the study. Therefore, the negative impact on psychological outcomes cannot be linked to the direct effect of COVID-19 disease; rather, it is more likely to be associated with the effects of social isolation and social deprivation or to health anxiety and intolerance of uncertainty. On this account, a recent study with the general population showed how, during the mandatory quarantine related to the COVID-19 outbreak in Italy, increased social isolation and social deprivation led to higher depressive symptoms and worse mental health [50]. During the lockdown, other factors could have negatively influenced cardiac patients’ psychological health. Reduced access to primary care as well as poor control of cardiovascular conditions and patients’ reluctance to seek medical help due to fear of contracting the virus during the lockdown have been reported [51]. The COVID-19 pandemic also impacted cardiac rehabilitation, leading to a temporary cessation of cardiac rehabilitation delivery or the implementation of new technologies such as home-based cardiac rehabilitation, telehealth, or online consultations in many cases [52]. Further studies are warranted to better understand the specific mechanisms that determine quarantine-related negative effects on psychological outcomes in cardiac patients.

The current findings should be interpreted in light of some possible methodological limitations. First, no healthy control group was available for the present study. The possibility that the effect of the quarantine on psychological outcomes did not differ between cardiac patients and the general population cannot be excluded. Nonetheless, a meta-analysis showed that the pooled prevalence of depressive symptoms in the general population during the quarantine was 25% [53]. In Italy, the presence of relevant depressive symptoms in the general population was between 17.3% [54] and 23.4% [55], and in one study, 15.4% of the sample reported very high depressive symptoms [2]. The present results showed that 30% of the cardiac patients were depressed after the quarantine, suggesting that cardiac patients might be at higher risk of developing clinically relevant depressive symptoms after quarantine than the general population. Second, this study used a relatively small sample size, which increased the risks for false-positives and limits the generalizability of the results. However, as previously suggested [56], how the sample was determined, all data exclusions and all data manipulations were disclosed. Power analysis showed that the sample size was adequate to identify small effect sizes. The effect sizes reported in the present study (*η*² for the BDI-II = 0.04; *η*² for the BDI-II cognitive = 0.06; *η*² for the BAI = 0.05) are consistent with the recommended minimum effect size representing a “practically” significant effect (*η*² = 0.04) [57]. Moreover, comparisons between the included patients and those who were not included showed no differences in age, sex, biomedical variables, or psychological variables between the groups. Patients who were included in the study had a slightly but significantly higher education level than patients who were not included in the study. Although a significant difference emerged between means, the average years in both groups correspond to upper secondary education in the Italian education system (ranging from 11 to 13 years of education). This suggests that the analyzed sample was representative of the population of cardiac patients after cardiac intervention. Finally, no measures of cardiovascular (e.g., angina pectoris, restenosis, heart failure) or functional (i.e., return to work rate, quality of life) outcomes were collected at reassessment. Future studies are warranted to evaluate the effects of COVID-19-related quarantine on cardiovascular and functional outcomes.

## Conclusion

The mandatory quarantine after the COVID-19 outbreak in Italy was linked to higher depressive, anxiety, and insomnia symptoms in cardiac patients eight months after cardiac intervention, while these symptoms usually tend to decline or stabilize in this period. This is of paramount relevance considering that depressive, anxiety and insomnia symptoms are linked to worse cardiac outcomes and a higher risk for mortality after cardiac intervention. The present results underline the importance of integrating and improving psychological assessments and interventions in cardiac rehabilitation programs.

## Supplementary information

Supplementary materials

## Data Availability

Deidentified participant data that underlie the results reported in this article (text, tables, figures, and appendices) and statistical analysis codes are available at https://osf.io/hps8j/.
