# Supplementary material for: The impact of COVID-19-related quarantine on psychological outcomes in patients after cardiac intervention: a multicenter longitudinal study
Source: Transl Psychiatry. 2022 Jun 6;12:235. doi: 10.1038/s41398-022-01984-0 (PMC9169951; doi:10.1038/s41398-022-01984-0)
Supplement: Supplementary file 1 — Supplementary materials [file 41398_2022_1984_MOESM1_ESM.docx]

**Supplementary materials**

**Table 1** Characteristics of patients included and not included in the study.

|  | **Included**  **(n = 149)** | **Not included**  **(n = 190)** | **t/χ^2^** | ***p*** |
| --- | --- | --- | --- | --- |
| **Demographic characteristics** |  |  |  |  |
| Age (year) | 61.98 (10.22) | 64.50 (10.45) | -2.23 | .026 |
| Gender, Male (N, %) | 129 (87) | 158 (83) | 0.51 | .474 |
| Education (years) | 12.73 (4.27) | 11.00 (4.36) | 3.67 | < .001 |
| BMI (Kg/m^2^) | 26.39 (3.30) | 26.45 (4.00) | -0.14 | .886 |
| **Type of cardiac intervention** |  |  | 0.0001 | .999 |
| Surgery (N, %) | 32 (21) | 41 (22) |  |  |
| PTCA (N, %) | 117 (79) | 149 (78) |  |  |
| Days from surgery | 30.01 (31.21) | 28.47 (20.84) | 0.52 | .605 |
| **Cardiac risk factors** |  |  |  |  |
| Hypertension (N, %) | 110 (74) | 132 (69) | 0.58 | .448 |
| Atrial fibrillation (N, %) | 37 (25) | 34 (18) | 2.03 | .155 |
| Diabetes (N, %) | 23 (15) | 35 (18) | 0.33 | .563 |
| Dyslipidemia (N, %) | 84 (56) | 114 (60) | 0.31 | .575 |
| CCI score | 2.64 (1.29) | 2.74 (1.21) | -0.71 | .478 |
| **Medications** |  |  |  |  |
| β-blockers (N, %) | 121 (81) | 157 (83) | 0.04 | .844 |
| Antihypertensive (N, %) | 52 (35) | 67 (35) | 0.0001 | .999 |
| Antiarrhythmics (N, %) | 21 (14) | 29 (15) | 0.02 | .883 |
| Anticoagulants (N, %) | 147 (99) | 185 (97) | 0.20 | .657 |
| ACE-inhibitors (N, %) | 70 (47) | 90 (47) | 0.0001 | .999 |
| Psychiatric drugs (N, %) | 6 (4) | 8 (4) | 0.0001 | .999 |
| **Biomedical and behavioral characteristics** | |  |  |  |
| Systolic Blood Pressure (mmHg) | 128.69 (15.12) | 128.20 (16.79) | 0.25 | .805 |
| Diastolic Blood Pressure (mmHg) | 77.60 (7.25) | 78.19 (8.77) | -0.58 | .562 |
| Walking (total minutes in the last week) | 219.21 (193.67) | 224.28 (286.64) | -0.19 | .847 |
| **Psychological variables** |  |  |  |  |
| BDI-II | 7.89 (6.15) | 8.36 (7.37) | -0.65 | .517 |
| BDI-II somatic | 5.74 (4.36) | 6.02 (4.79) | -0.57 | .571 |
| BDI-II cognitive | 2.15 (2.56) | 2.36 (3.18) | -0.67 | .500 |
| BAI | 8.45 (7.27) | 8.22 (7.01) | 0.29 | .770 |
| SCI | 24.25 (6.02) | 22.53 (7.23) | 2.38 | .018 |

*Note*: Data are *M (SD)* of continuous and *N (%)* of categorical variables. BMI = body mass index; CCI = age-adjusted Charlson Comorbidity Index; ACE- inhibitors = angiotensin-converting-enzyme inhibitors; BDI-II = Beck Depression Inventory II; BAI = Beck Anxiety Inventory; SCI = Sleep Condition Indicator.

**Table 2** Logistic regression model predicting the reassessment prevalence of depression.

| **Model 1** |  |  |  |  |  |  |  |  |
| --- | --- | --- | --- | --- | --- | --- | --- | --- |
| **Predictor** | **β** | **SE** | **Z** | ***OR*** | ***p*** | **Deviance** | **AIC** | **R²McF** |
|  |  |  |  |  |  | 113.50 | 117.50 | 0.23 |
| Intercept | -2.10 | 0.28 | -7.41 | 0.12 | < .001 |  |  |  |
| Prevalence of depression at assessment | 3.01 | 0.56 | 5.38 | 20.36 | < .001 |  |  |  |
| **Model 2** |  |  |  |  |  |  |  |  |
| **Predictor** | **β** | **SE** | **Z** | ***OR*** | ***p*** | **Deviance** | **AIC** | **R²McF** |
|  |  |  |  |  |  | 105.80 | 111.80 | 0.28 |
| Intercept | -2.96 | 0.49 | -5.99 | 0.05 | < .001 |  |  |  |
| Prevalence of depression at assessment | 3.00 | 0.59 | 5.06 | 20.18 | < .001 |  |  |  |
| Group (Quarantine vs. Control) | 1.44 | 0.55 | 2.61 | 4.20 | .009 |  |  |  |
